# Energy Landscapes of Model Knotted Polymers

**DOI:** 10.1021/acs.jctc.5c01005

**Published:** 2025-08-14

**Authors:** Tongfan Hao, Yinghao Ge, Mark A. Miller, Agustin L. N. Francesco, David J. Wales

**Affiliations:** † School of Materials Science and Engineering, 12676 Jiangsu University, 301 Xuefu Road, Zhenjiang 212013, China; ‡ Yusuf Hamied Department of Chemistry, 506396 University of Cambridge, Lensfield Road, Cambridge CB2 1EW, U.K.; § Department of Chemistry, 3057 Durham University, South Road, Durham DH1 3LE, U.K.

## Abstract

The energy landscape of a model knotted ring polymer, consisting of 100 Lennard-Jones particles connected by harmonic springs, is extensively characterized for three topologies. Basin-hopping global optimization with unrestricted perturbation moves for the geometry can efficiently locate the global minimum of the topologically unconstrained energy landscape. We show that an isotropic radial potential to expand the structure provides a robust way to assign the crossing number and topology of each configuration. The radial potential also provides a way to propose more efficient geometrical perturbations for basin-hopping that preserve the topology, which should be generally applicable to molecular and soft matter systems. All three topologies exhibit multifunnel landscapes with a wide range of relaxation time scales, clearly visible in first passage time distributions. The global minimum corresponds to a particularly favorable, symmetrical packing, which produces a pronounced heat capacity peak. In contrast, the unknotted ring topology supports alternative low-energy minima that constitute remarkable kinetic traps and associated broken ergodicity. Such features may present opportunities for materials design.

## Graphical abstract

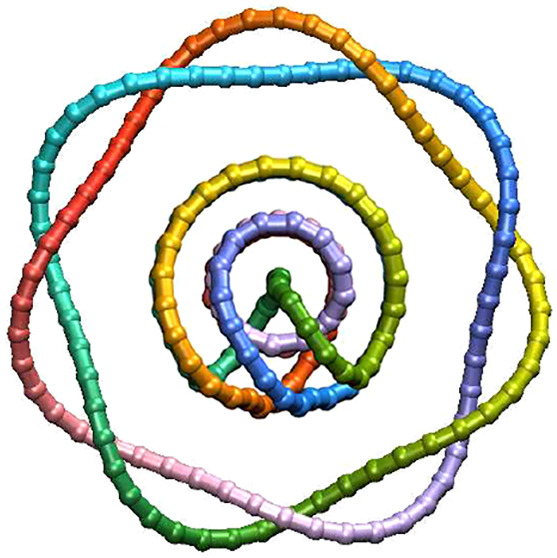

## I Introduction

The ability of a molecule to self-organize reliably into a particular structure is encoded in the underlying potential energy surface.^1^ When this surface is described in terms of local minima and the transition states that connect them, a potential energy landscape with a single funnel provides a solution to Levinthal’s paradox,^2^ with a well-defined free energy minimum that is kinetically accessible over a range of temperature or total energy. For a suitable order parameter that reports on the separation from the global minimum, a single-funnel potential energy surface corresponds to a funnelled free energy landscape.^3–8^ For a biomolecule this organization supports kinetically convergent pathways leading to the native state.^9^

In contrast, multifunnel landscapes have competing structures separated by high barriers,^10,11^ corresponding to broken ergodicity and frustration.^12^ Such features can be exploited, for example to design a molecular switch, which would require a double-funnel landscape where the relative stability can be tuned by an external parameter, such as the temperature.^7,10,13,14^ Multifunnel landscapes have been characterized for a number of evolved multifunctional biomolecules,^15^ while glassy landscapes represent the extreme limit with an exponentially large number of local funnels corresponding to amorphous states.^16–21^ For single-funnel landscapes, locating the global minimum is usually straightforward, both in experimental systems, and in calculations. In contrast, exploration of multifunnel landscapes is hindered by the high barriers, and manifests in terms of broken ergodicity, corresponding to multiple heat capacity features and relaxation time scales.^10,11,22^ Visualizing the landscape using disconnectivity graphs^13,23^ can provide direct insight into the organization and guide analysis of structure, dynamics, and thermodynamic properties.

As a representative polymer system that supports alternative topologies, knotted ring polymers provide an attractive model for investigating how observable properties are encoded in the energy landscape. Here, we examine the landscapes for ring polymers of particles connected by harmonic springs, with nonbonded interactions described by a Lennard–Jones potential.^24^ When a linear polymer chain possesses sufficient length and flexibility, it almost inevitably becomes entangled.^25–28^ Given a formal closure of the chain to connect the ends,^29,30^ such entanglements constitute topological knots. Multiscale studies, ranging from macroscopic ropes to DNA, proteins, and synthetic polymers, have demonstrated that the presence of knots can significantly influence the dynamics, chemical reactivity, biological functions, and mechanical properties of polymer systems.^31–38^ For example, at the macroscopic scale, knots can reduce the tensile strength of a rope by approximately 50%; at the molecular level, a trefoil knot can lower the rupture force of a polymer backbone from more than 5.6 to 2.9 nN, and accelerates chain scission by a factor of more than 2.6 under tension.^39^ Naturally occurring knots in DNA and proteins play important roles, regulating gene replication, transcription, and enzymatic activity, as well as facilitating processes such as viral DNA injection and nanopore sequencing.^40–42^ Recent mechanochemical experiments further reveal that molecular knots represent some of the most reactive mechanophores in polymer systems.^39^ Hence, knotted polymers serve not only as ideal model systems for investigating topological effects and nonequilibrium mechanical behavior at the molecular scale, but may also provide new design principles for developing smart mechanoresponsive materials and high-performance structural polymers.

The significant role of knotted structures in practical applications has also motivated computer simulations^43–51^ and theoretical analysis of their underlying physical properties. De Gennes was among the first to propose that, under specific conditions, knotted regions can adopt metastable, tightly compact conformations, which may substantially alter the relaxation behavior of polymer chains.^52^ Subsequently, Grosberg and Rabin established the well-known tube model framework, demonstrating that the competition between bending energy and configurational entropy can drive knot localization and the formation of metastable states.^53^ Building on this theory, Dai and co-workers systematically extended and validated the model through off-lattice simulations, confirming the existence of metastable knot sizes.^54^ They further revealed a counterintuitive regulation of knot size by varying the strength and range of intrachain interactions^55^ and proposed an improved tube model capable of quantitatively predicting the geometric and energetic features of knotted conformations.^56^ Marenz and Janke used Monte Carlo simulations to investigate knotted structures in semiflexible open polymer chains. Since a closure is applied to individual snapshots, the topology is not conserved during the simulation, making it particularly interesting to analyze the types of knots that arise. These authors evaluated the Alexander polynomial and used it to construct a topological order parameter, which proved effective in building a pseudophase diagram. They also noted that torus knots are prevalent,^57^ consistent with studies on DNA^58^ and self-assembled knots.^25,59,60^ In the present work we have also concentrated on two torus knots (as well as the unknot, which is a trivial torus knot), in closed chains with fixed topology.

Building upon previous models, recent studies have increasingly focused on incorporating additional physical interactions, particularly electrostatics and chain stiffness.^61–64^ This research has shown that electrostatic interactions can significantly influence knot formation and stability, and may even induce metastable conformations and responsive structural transitions.^65–67^ Moreover, chain stiffness also has a clear impact on the spatial distribution of knots, especially in flexible-rigid block copolymers, where knots tend to localize near the interface between the two segments.^68–70^ These results collectively suggest that incorporating electrostatic potentials (such as Debye–Hückel screening) or angular stiffness terms into the modeling framework is feasible. In the present contribution, we focus on a model of ring polymers that contains the minimal physical interactions required, to serve as a reference for future work. Electrostatic and rigidity effects are both compatible with the framework we employ, and analysis of more specific models for knotted macromolecules, such as DNA and polyelectrolytes, is an interesting prospect.

Previous work has produced substantial progress in the theoretical understanding of knotted polymers. Nevertheless, compared to other polymer topologies, the theoretical investigation of knotted polymers remains at a relatively early stage, particularly in terms of how distinct knot topologies organize within the potential energy landscape and give rise to multifunnel features. Part of our goal in the present contribution is to show how such properties are encoded in the landscape for a single ring polymer using a generic potential energy function that is intended to capture the essential physics. Some hint of the possible complexity can be found in an earlier study of 7-particle polymer landscapes and properties,^71^ and in previous work on clusters of dipolar particles, which can also favor chain-like structures with knotted topologies.^59,72^ The present contribution shows how this complexity is realized for different ring-polymer topologies, highlighting the associated global kinetic and thermodynamic signatures.

We investigate model ring homopolymers of 100 particles with selected knotted topologies, which are examples of knots with particular crossing numbers. The crossing number is a fundamental topological invariant that provides one measure to quantify the complexity of a knot. It is defined as the minimum number of crossings observed in any regular planar projection of the knot. For a given knot type, the crossing number represents the smallest number of over/under intersections that cannot be eliminated through continuous deformation without cutting or passing through the strand.^73^ For the ring polymer of 100 particles, we denote the unknot by K_0_, the trefoil (3_1_ in the Rolfsen notation^74^) by K_3_1__, the 7_1_ torus knot by K_7_1__, and all three generically as K^100^. Our principal aim is to investigate the structure and connectivity of low-energy isomers for the three different topologies. We first employ basin-hopping global optimization,^75–77^ followed by discrete path sampling^78,79^ to construct kinetic transition networks,^80–82^ which consist of local minima and the transition states that connect them. The model potential used to describe the ring polymers allows chains to cross, and hence interconversion of knots with different topologies. However, the corresponding barriers are very high compared to the energy scales involved for rearrangements that conserve the topology. Hence, we can treat the landscapes for different topologies separately, and compare their organization.

We have also considered calculations to investigate the packing of low-energy minima. In particular, we subjected the structures to applied forces that expand the structure without changing the topology. This analysis was suggested by an expansion scheme that we developed to allow a straightforward identification of the crossing number and knot type, which is difficult for the packed chains. It was also used to propose more efficient geometry perturbations for moves proposed in basin-hopping global optimization, as described in Section 2.2. The complexity of the packed structures can be quantified in terms of the number of crossings for randomly generated projections onto the planes, as described in Section 2.4. The corresponding order parameters provide useful additional insight. The multifunnel landscapes we have characterized for K^100^ knots originate from alternative packings of the chain, but examination of representative minima from competing funnels does not yield a simple description of the structural organization. However, order parameters based on complexity measures calculated from projected chain crossings and topological analysis can discriminate between some of these funnels, as described in Section 3. We considered other possible order parameters, including the principal moments of inertia and the log product of positive Hessian eigenvalues. These results are omitted for brevity: instead we focus on two particular topological order parameters that successfully provide additional insight into the organization of the landscape, namely the winding number (Section 2.6) and the writhe (Section 2.5).

## II Methods

### II.I Potential

We study the structural and energetic properties of knotted ring polymers using a coarse-grained bead–spring model. The nonbonded interactions between all bead pairs (excluding directly bonded particles) are described by a Lennard–Jones (LJ) potential

1 $$U_{\mathrm{LJ}}\left(r\right)=4\varepsilon \left[{\left(\frac{\sigma }{r}\right)}^{12}-{\left(\frac{\sigma }{r}\right)}^{6}\right]$$

where *r* is the distance between two beads, while ε = 1 and σ = 1.888 define the LJ energy and length scales, respectively. The bonded interactions between adjacent beads are described by a harmonic potential

2 $$U_{\mathrm{bond}}\left(r\right)=\frac{1}{2}K{\left(r-r_{0}\right)}^{2}$$

where *K* = 1035.8 is the spring constant, and *r*_0_ = 1 is the equilibrium bond length. K and σ were chosen to match the effective force constant and length scale used in previous work where finite extensible nonlinear elastic (FENE) bonds^83,84^ were employed. This representation is a popular choice for large-scale polymer simulations, but is inconvenient for our methodology where large geometry perturbations are required for efficient sampling. We have compared the full landscape obtained for the original FENE bond representation with the harmonic bonding potential for K_3_1__^100^. The stationary points and connectivity of the local minima are practically the same and the corresponding disconnectivity graphs are almost indistinguishable. Hence our results are directly comparable with previous work.^83,84^

The relatively large force constant in eq 2 corresponds to stiff bonds, which ensure stable connectivity and cause the distance between adjacent beads to be quite uniform in all the local minima. Hence, ε, which specifies the strength of the nonbonded attraction in eq 1, is the main parameter to set the relevant energy scale of the model. As a result, small variations in ε will simply scale the vertical energy axis in the disconnectivity graphs, and thermodynamic properties are practically unchanged if expressed in units of ε. We have tested the effect of parameter changes using a key pathway of interest for K_7_1__^100^ by systematically varying ε and σ. The results are summarized in the Supporting Information, where we see that changes in ε by ±15% simply shift all the stationary points almost uniformly, and so the organization of the landscape is unaffected. Not surprisingly, changes in σ have a greater effect, and variations beyond one or two percent perturb the landscape significantly.

The landscapes will also depend on the chain length, and we plan to investigate these effects in future work. Preliminary surveys suggest that some of the organization is preserved if the chain length changes by a few particles. However, longer chains of 200 particles are likely to produce even more frustrated landscapes than those described here, with a larger number of significant local funnels and higher barriers associated with reorganization of more particles.

### II.II Landscape Exploration

All the calculations for global optimization and creation of kinetic transition networks are based on geometry optimization procedures, which have been employed in many previous studies and reviewed elsewhere.^15,85,86^ For basin-hopping global optimization^75–77^ we use the GMIN program,^87^ which includes many variations on the basic algorithm of stepping between local minima obtained by perturbing the current structure in a chain.

To construct kinetic transition networks we use the OPTIM program,^88^ with connection attempts between different local minima organized in parallel by PATHSAMPLE.^89^ The choice of connection attempts can be adjusted to locate the fastest pathway between selected minima,^90,91^ or remove artificial frustration in the landscape to seek connections with lower barriers between low-lying minima.^91^ Harvesting these databases corresponds to the discrete path sampling approach.^78,79^ Transition state candidates are located using the doubly nudged^92,93^ elastic band^94–97^ method, and refined by hybrid eigenvector-following.^98–100^ The minima connected by each transition state are determined from the two approximate steepest-descent pathways, and gaps in a connection profile are filled in using the missing connection algorithm to propose the next round of searches.^90^

The potential energy is invariant to the *N* cyclic permutations of bead coordinates for K^*N*^, and to *N* permutations with the reverse sequence. The corresponding local minima and associated transition states are lumped together in the database and the associated disconnectivity graph visualizations discussed below. For all connection attempts between local minima the structures were aligned to minimize the Euclidean distance with respect to translation, rotation, and the full set of permutations. Here we employed an iterative procedure, since the distance minimization is not deterministic, with the shortest augmenting path algorithm^101^ embedded in the loop.^102^ This is a standard procedure available in the GMIN, OPTIM, and PATHSAMPLE programs, and exploiting the permutational symmetry significantly enhances the exploration of the landscape.

The usual procedure for assigning the crossing number and knot type for a given structure uses random two-dimensional (2D) projections, as described in Section 2.4. When applied to the compact structures observed for low-energy local minima in the K^100^ ring polymers we found that this approach often failed because there were too many crossings. However, we were able to apply the algorithm to check our visual inspection, after expanding the structure. We first considered a pulling potential between selected pairs of beads, as coded in GMIN and OPTIM. This potential has previously been employed to analyze the landscape for two proteins subject to a static pulling force.^103^ Choosing two beads well separated in the ring, and systematically increasing the pulling force constant, it is straightforward to expand the structure without changing the topology, and the crossings can then simply be counted directly. The need to choose attachment points can be avoided by applying a radial potential from the center of coordinates, **r**_c_

3 $$V_{\mathrm{radial}}=\frac{k}{2}\sum_{\alpha =1}^{N}|r_{\alpha }-r_{c}{|}^{p},,\mathrm{where},r_{c}=\frac{1}{N}\sum_{\alpha =1}^{N}r_{\alpha }$$

Here **r**_α_ is the position vector of bead α, *k* is the force constant, and *p* is an adjustable power. We changed *k* systematically until the packed structure expanded, which works straightforwardly for *p* = 1 and 2. The radial potential was already available in GMIN and OPTIM, and has previously been used to study compression in atomic clusters^104^ for *k* > 0. Here we simply considered *k* < 0 and increased this parameter in magnitude gradually. A value of *k* = −15 with power *p* = 1 was sufficient to expand all the local minima we encountered in this study for classification; expansion trajectories are provided as movies in the Supporting Information. The pulling and expansion approaches employed in the present work are different from a previous formulation that considered repelling particles,^105^ because the applied fields do not involve two-body terms. The barriers for chain crossing are much larger than any barriers encountered in the expansion process, and we found that the pulling and radial potentials enabled us to identify the crossing number and topology efficiently; examples are shown in Figure 1. This expansion procedure also works for the linked knot and composite knots that we have tested. Examples for the K_3_1__^100^ linked dimer and the K_3_1_#3_1__^100^ composite knot are shown in the Supporting Information; we will investigate such systems in future work. On varying *k* for K_3_1__^100^ and K_7_1__^100^ we observe two possible expanded morphologies with either localized crossings or a more even distribution. The structures with localized crossings appear when the expansion potential is stronger. Localized and more expanded structures are illustrated for K_3_1__^100^ and K_7_1__^100^, respectively, in Figure 1. The alternatives are shown in the Supporting Information.

**Figure 1:**
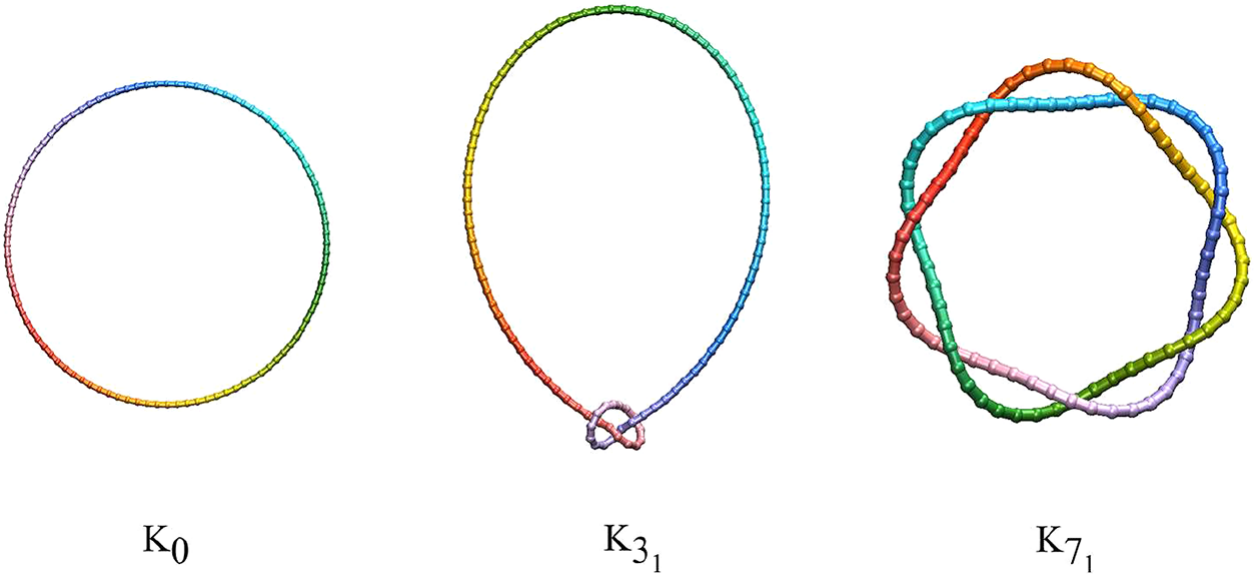
Examples of expanded structures obtained by applying the radial expansion potential defined in eq 3. Alternative expanded structures identified for K_3_1__^100^ and K_7_1__^100^ are illustrated in the Supporting Information.

The same radial potential was employed in basin-hopping global optimization to propose geometrical perturbations that conserve the topology. Here we applied a compressive potential at the start of each local minimization and turned it off when the root-mean-squared gradient fell below a selected threshold (values around 0.01 units were found to work). This compression produces a geometrical perturbation that can move the configuration to the basin of attraction^1,106^ of a neighboring local minimum without changing the topology.

### II.III Thermodynamics and Kinetics

Further insight into how observable kinetic and thermodynamic properties are encoded in the landscape were obtained by postprocessing calculations for the kinetic transition networks obtained from discrete path sampling. To calculate the heat capacity we write the total partition function as a sum over all the local minima using the superposition approach.^1,107,108^ The vibrational densities of states were obtained for each minimum in the harmonic approximation using analytical second derivatives of the potential energy. This approach is explicitly ergodic by construction, and includes landscape anharmonicity and entropy^7,109^ but not well anharmonicity. Further details are available in reviews.^1,15,85,86^

A direct measure of the relaxation properties was obtained by calculating the mean first passage time (MFPT) to the lowest minimum starting from all the other local minima using graph transformation.^110,111^ This method provides a numerically robust approach, even for landscapes that support deep kinetic traps with very slow transition rates and ill-conditioned kinetic matrices, as reviewed elsewhere.^112^ These calculations require the minimum-to-minimum rate constants that appear in the corresponding master equation, which were obtained from transition state theory^113–117^ using harmonic densities of states, consistent with the thermodynamic analysis. This approximation is often employed for treatment of global dynamics,^118–122^ and is appropriate to demonstrate the appearance of multiple relaxation time scales for the frustrated^12^ landscapes considered here.

We have also calculated first passage time (FPT) distributions, which contain a wealth of information beyond the mean value.^123–126^ In particular, the FPT, denoted as θ in Figure 5, directly reveals multiple relaxation time scales associated with multifunnel landscapes.^22,127,128^ The FPT calculations are significantly more involved than for the MFPT, and require eigendecomposition of the ill-conditioned rate matrix that appears in the master equation for the kinetic transition network.^22,126–128^ The knot landscapes proved to be particularly challenging, and required partial graph transformation, which uses additional coarse-graining.^129^ By judicious choice of the states that are renormalized, it is possible to reduce the dimensionality of the problem while conserving the FPT to good accuracy.^129^ It was then possible to apply a full eigendecomposition using LAPACK routine DGEEV, and treating the slowest relaxations separately if the corresponding eigenvectors were not determined properly. We can use the MFPT from graph transformation, and the normalization condition, to deduce the position and height of the FPT peak corresponding to the missing mode.^130^ It was then possible to characterize multipeak FPT distributions at temperatures above the main heat capacity peak.

### II.IV Knot Identification

To identify the knot type of a given structure, we employed a Jones polynomial-based topological classification method via random 2D projections.^73^ Specifically, each knot configuration was subjected to 10,000 random rotations, and the rotated configurations were projected onto the *xy* plane. Crossings between chain segments were identified in each projection, and the over/under relationships at crossing points were determined by comparing the *z*-coordinates of the intersecting segments. Among all projections, the one with the minimal number of crossings was selected. The corresponding crossing sequence was then passed to a Jones polynomial evaluation program, which recursively computes the polynomial using the skein relation.^73^ As a topological invariant, the resulting Jones polynomial was compared against known reference polynomials to determine the knot type of the structure. This algorithm often failed for the compact structures of low-lying K^100^ local minima, because there are so many crossings. However, it worked for the expanded structures obtained using a pulling or radial potential that should conserve the topology, as discussed in Section 2.2. We therefore used this procedure to check our visual assignments and the preservation of topology.

### II.V Calculation of the Writhe

To characterize the geometrical complexity of the knot configurations, we calculated both the total number of crossings and the writhe from two-dimensional projections of the three-dimensional (3D) structure. In each projection, the number of crossings is defined as the total count of intersecting points between oriented segments of the chain, regardless of over/under status or direction. To compute the writhe, we followed the standard convention from knot theory:^73^ a direction is first assigned along the loop, and each crossing is classified as either positive or negative depending on the relative orientation of the overpassing and underpassing strands. A crossing is considered positive when the overpassing strand runs from left to right while the underpassing strand runs from bottom to top; otherwise, it is negative. The writhe is then calculated as the algebraic sum of +1 or −1 for all crossings.

This analysis also involved subjecting the 3D knot structure to 10,000 random rotations, with each rotated configuration projected onto the *xy* plane. In each projection, we identify all segment intersections and determine the over/under relationship by comparing the *z*-coordinates at the crossing points. Unlike knot type identification, which only considers the projection with the minimal number of crossings, the total number of crossings and the writhe are calculated as the mean and standard deviation across all projections, providing a more comprehensive measure of the knot’s average topological complexity for varying orientations.

### II.VI Winding Number

To characterize the topological behavior of individual closed loops in the projection plane, we calculated the winding number (WN), which quantifies how many times a closed curve turns around a specified reference point. In this study, the reference point was chosen as the center of coordinates defined in eq 3, **r**_c_, and each particle position **r**_*i*_ was transformed into a vector: **v**_*i*_ = **r**_*i*_ – **r**_c_, which was then projected onto the *xy* plane, giving **v**_*i*_^′^ = **v** – (**v**·**k**) **k**, where **k** is the unit vector parallel to *z*. The winding number was computed by summing the signed angular displacements between successive vectors **v**_*i*_^′^ and **v**_*i*+1_^′^. The incremental angle χ_*i*_ at each step was calculated as

4 $$\chi_{i}=\mathrm{atan}2\left(\left[v_{i}^{\prime }\times v_{i+1}^{\prime }\right]\cdot k,v_{i}^{\prime }\cdot v_{i+1}^{\prime }\right)$$

The first argument is the *z* component of the cross product of projected position vectors, which is negative or positive for clockwise or counterclockwise rotations, respectively. The scalar product in the second argument is used to define the absolute value of the angle between the two vectors. The total winding number for a given projection is then defined as

5 $$\mathrm{WN}=\left|\frac{1}{2\pi }\sum_{i=1}^{N}\chi_{i}\right|$$

Consistent with the treatment of total crossings and writhe, the final winding number is reported as the average over 10,000 randomly rotated projections.

## III Results

The lowest minima found for the three K^100^ knots are listed in Table 1. To locate the global minimum, irrespective of topology, we simply employed basin-hopping^75–77^ with a relatively large maximum coordinate perturbation comparable to the separation of the bonded particles. In contrast, searches for the lowest minimum with a particular topology require steps that do not change the crossing number. We found that a maximum perturbation of 0.69 was small enough to prevent chains from crossing during global optimization, but makes these searches less efficient. However, applying a radial compression and then releasing it can move the configuration between local minima without changing the crossing number. We therefore adopted a new procedure to search for the lowest K_0_ and K_3_1__ structures, using compression for every coordinate perturbation as described in Section 2.2. Here we applied the radial potential defined in eq 3 with a harmonic term and positive force constants (around 5 units).

**Table 1:** Lowest Minima from the Three K^100^ Landscapes

knot	energy
K_0_	–738.992818
K_3_1__	–738.238236
K_7_1__	–760.585645

The three landscapes we have explored in the present contribution all support low-lying minima separated by high barriers, as described in detail below. Basin-hopping can locate low-lying minima under such conditions because the steps do not need to satisfy detailed balance, and moves are accepted and rejected based on the energies of local minima, not on instantaneous values. To construct a kinetic transition network we progressively connect local minima via transition states. The corresponding geometry optimization techniques are largely agnostic to the barrier heights involved, which enables us to predict global thermodynamic and kinetic properties of the landscape, subject to well-defined approximations.

We located low-energy minima for K_0_ that proved particularly difficult to connect. To produce the corresponding pathways we expanded the two target minima to a common structure, again using the radial potential employed for topology assignment, saving configurations along these relaxation trajectories. One of these trajectories was then reversed, and configurations were chosen at regular intervals from the combined set for initialization of a double-ended connection search (Section 2.2). We repeated these connections between the lowest three funnel bottoms and obtained multistep paths of similar length, which were all added to the database of stationary points. It is certainly possible that shorter pathways or lower overall barriers exist, but judging from the complexity of the changes in packing, it seems unlikely that they could be significantly simpler.

The heat capacity *C*_*V*_ is illustrated for the three landscapes in Figure 2. It is noteworthy that *C*_*V*_ for K_7_1__ has the most pronounced peak, in agreement with the structure of the landscape, as discussed below. The peaks correspond to the finite system analogue of a phase transition, and can be assigned to local minima via changes in the occupation probabilities at the corresponding temperature.^131^ For *C*_*V*_ calculated using harmonic densities of states (Section 2.3) this assignment is particularly clear: we can decompose the function into sums over local minima with positive and negative occupation probability gradients, *g*_γ_(*T*) = ∂*p*_γ_(*T*) /∂*T*

6

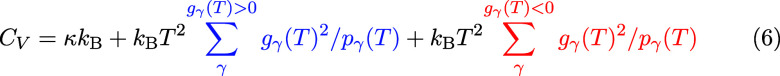

where κ = 3*N* – 6 is the number of vibrational degrees of freedom, *k*_B_ is Boltzmann’s constant, and *T* is temperature. The colors are chosen to match the highlighting scheme employed for the disconnectivity graphs in Figure 3. By sorting the contributions from the two sets we can immediately identify the key minima, and truncate the sums in eq 6 at a given fraction of the total. We can then highlight these minima in disconnectivity graphs.^13,23^ In this representation branches terminate at the potential energies of local minima, which are connected at the lowest energy where they can interconvert, with energy increasing on the vertical axis. This construction is based on the local minima and transition states of the kinetic transition network, as mentioned in the Introduction. The minima are arranged on the horizontal axis so that the lowest members appear in the middle of each funnelled region.

**Figure 2:**
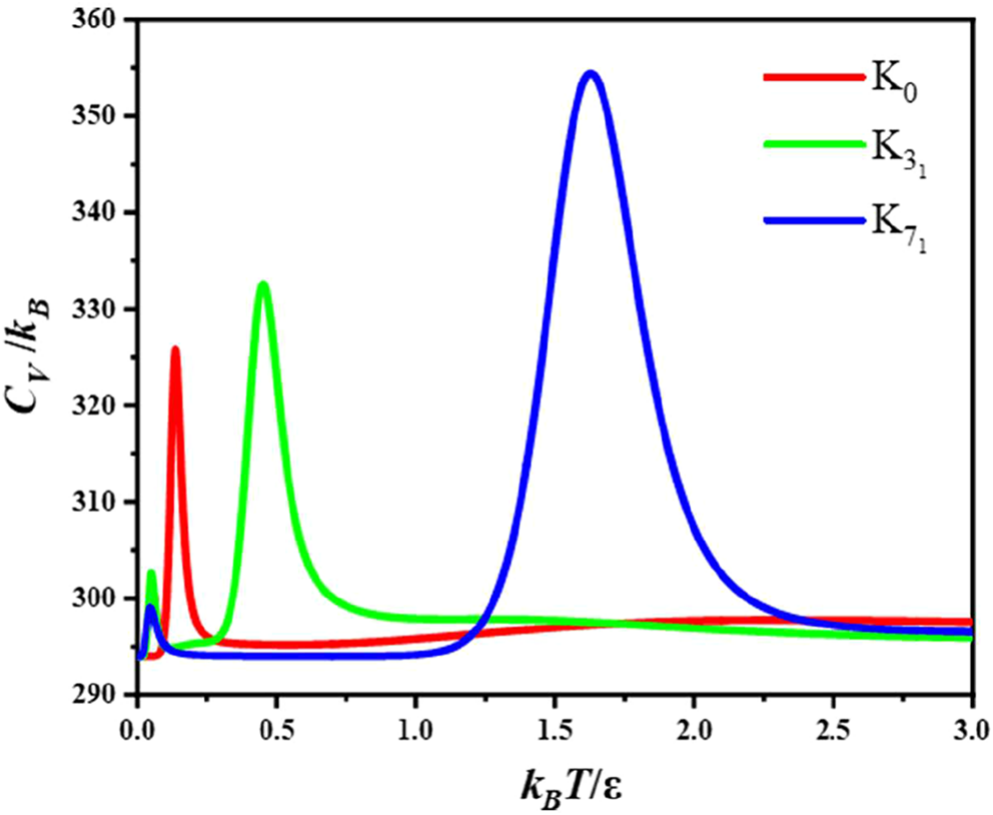
Heat capacity *C*_*V*_/*k*_B_ for the K_0_, K_3_1__, and K_7_1__ landscapes.

**Figure 3:**
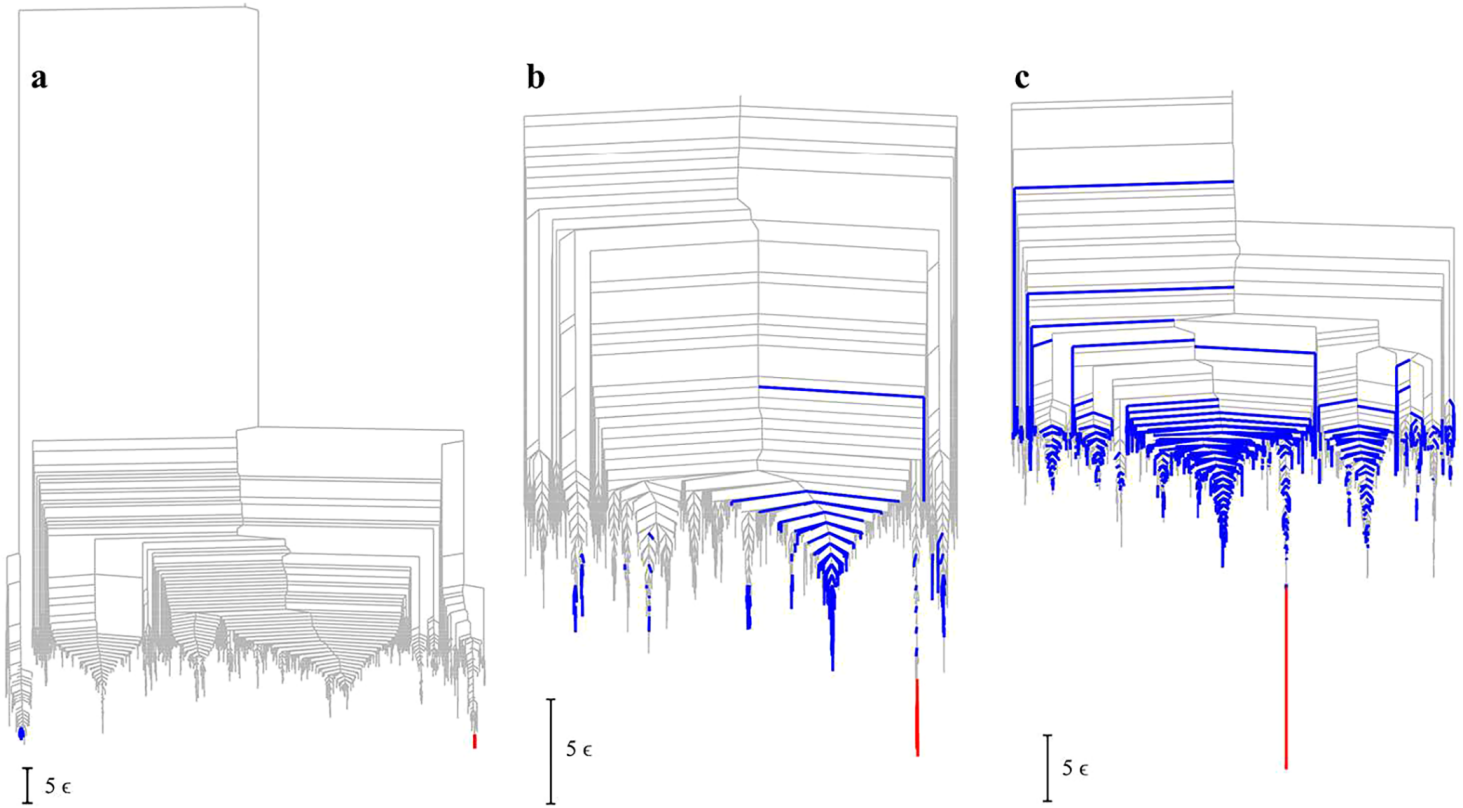
Disconnectivity graphs colored using the heat capacity breakdown^131^ for the (a) K_0_, (b) K_3_1__, and (c) K_7_1__ landscapes. Minima marked in **red** and **blue** have negative and positive temperature derivatives for the occupation probability at the selected temperature. Here the chosen temperatures are 0.139, 0.452, and 1.628 in energy units, corresponding to the largest peaks in *C*_*V*_ in Figure 2. In each case we only include connected minima from the lowest 3000 of each topology.

Figure 3 shows the landscapes colored according to the contributions to the largest, high temperature peak in *C*_*V*_. In each case the transition is from a small number of minima in red to a larger number of higher energy minima in blue with higher entropy. K_3_1__ and K_7_1__ also have a low temperature feature (Figure 2), and fewer than ten minima are needed to reproduce 99% of *C*_*V*_ for these transitions. The higher temperature transition is of more interest here, because it reflects the organization of a wider range of the landscape and includes some of the multifunnel structure. K_0_ has a very deep kinetic trap, which produces the principal heat capacity peak at a relatively low temperature. We comment further on the organization of this landscape below. The transition for K_7_1__ involves a larger number of higher energy minima with a significant change in potential energy and entropy. However, the higher energy minima are still compact structures, so this feature is not a melting transition. Rather, it corresponds to a change in diversity from the unique, low energy minimum, to a set of relatively low-energy structures that cannot match the favorable packing of the global minimum. More of these minima, and a higher temperature, are needed for the corresponding entropy change to compensate for the potential energy difference.

To investigate the kinetic accessibility of different regions in the landscape, Figure 4 presents the same disconnectivity graphs with branches colored according to the mean first passage time (MFPT Section 2.3) from each local minimum to the lowest minimum. The color gradient from red to blue indicates increasing MFPT values, i.e., a transition from kinetically accessible minima to kinetically isolated ones with longer relaxation times. These kinetic features complement the thermodynamic behavior presented in Figures 2 and 3.

**Figure 4:**
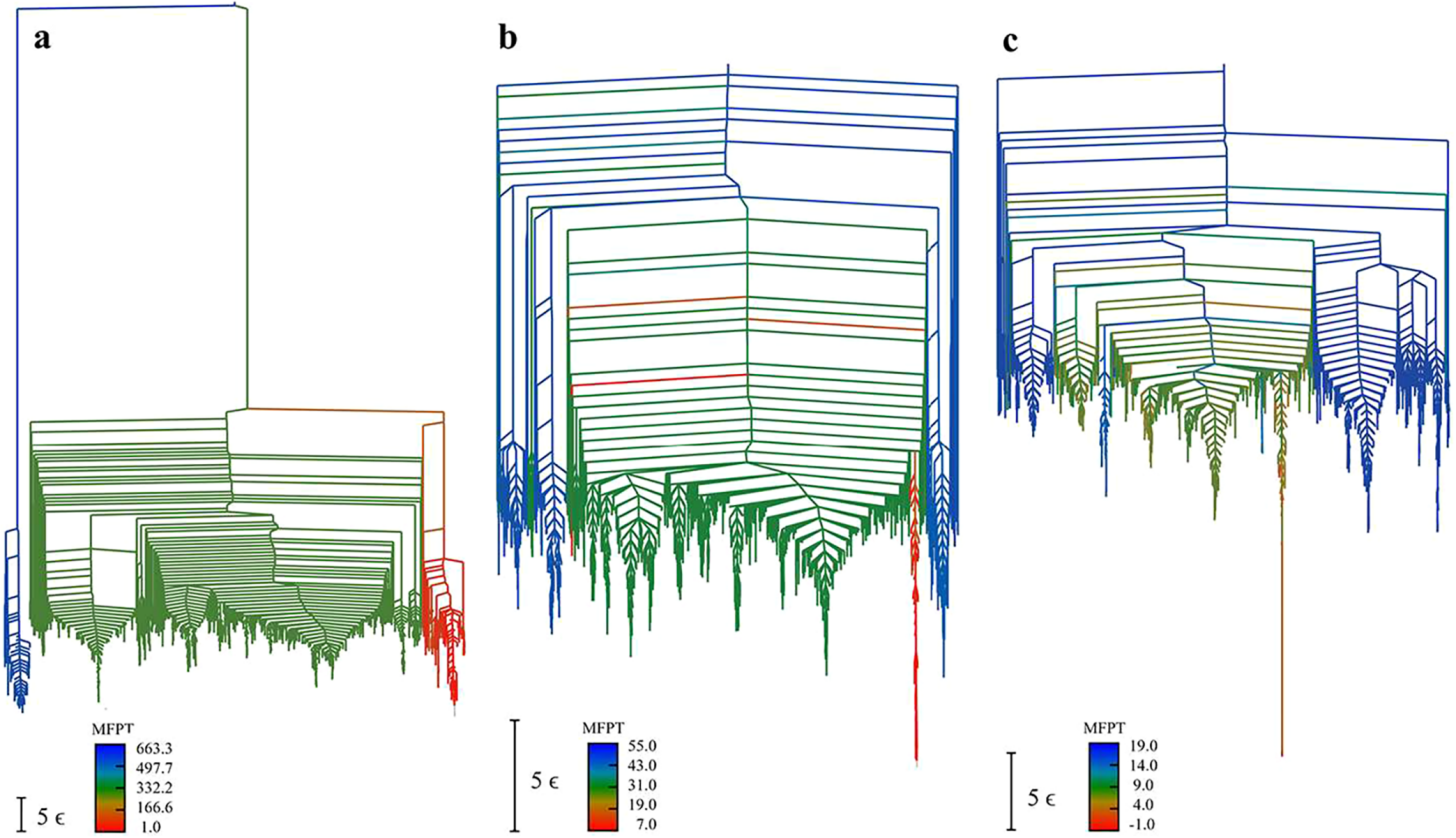
Disconnectivity graphs colored according to the MFPT to the global minimum for (a) K_0_, (b) K_3_1__, and (c) K_7_1__. The MFPT values are represented using a color gradient with red indicating shorter times and blue representing longer times. The temperatures for the MFPT calculations are 0.16, 0.452, and 1.628 in energy units, respectively. The same minima are included as in Figure 3.

For K_0_, the red regions in the disconnectivity graph are concentrated within the global minimum funnel, indicating that these low-energy minima are well connected kinetically and can interconvert with the global minimum quite rapidly, resulting locally in high overall reorganization efficiency. The next set of minima that appear in green have intermediate MFPT values that are very long compared to the slowest processes identified for the other two topologies. K_0_ exhibits a remarkably deep alternative packing. The temperatures chosen for the MFPT analysis are the same as for the heat capacity breakdown, except that a slightly higher value was required for K_0_. The graph transformation procedure^110,111^ is numerically very robust, but the kinetic trap for this landscape is so deep that a temperature of 0.16 energy units was required to prevent underflow in the calculations. The calculated barrier for the fastest path from this trap to the lowest minimum is over 700 *k*_B_ *T* at the temperature of the heat capacity peak, which constitutes an extreme example of broken ergodicity. The fastest path between these minima, calculated using Dijkstra’s algorithm^132^ with suitable edge weights,^78^ has around 630 transition states. It features a collective reorganization and a gradual change in energy rather than any well-defined rate-determining step. The paths where we expand local minima to deduce the crossing number exhibit twisting of the ring for K_0_, and this effect may be the cause of the high barriers observed in this landscape (see the corresponding movie in the SI).

Of the 819,514 distinct local minima characterized for the three knot topologies, only 61 have nontrivial point group symmetry. This set includes the global minimum for K_7_1__, which has a *C*_2_ axis, consistent with the principle of maximum symmetry.^1^

For K_3_1__, the minima colored red, which can access the global minimum on relatively short time scales, are nearly all in the same funnel. There are some relatively deep funnels that can only access the lowest minimum on longer time scales. This structure again indicates significant kinetic separation and potential trapping, but not as extreme as for K_0_. For K_7_1__, the time scale for escape from the lowest traps is faster than for the deepest traps in K_3_1__ at the chosen temperatures. We expect relaxation to the global minimum to be most efficient for K_7_1__, intermediate for K_3_1__, and strongly frustrated for K_0_. These kinetic results correlate with the progression of the principal heat capacity peak in Figure 2.

We analyzed the relaxation dynamics in more detail by calculating full first passage time (FPT) distributions to the lowest minimum. These calculations are significantly more involved than the MFPT analysis, and required further analysis of the kinetic transition network, as described in Section 2.3. Some selected results are shown in Figure 5 for one representative starting minimum to highlight the existence of multiple relaxation time scales; similar results are obtained when we start from alternative minima.

**Figure 5:**
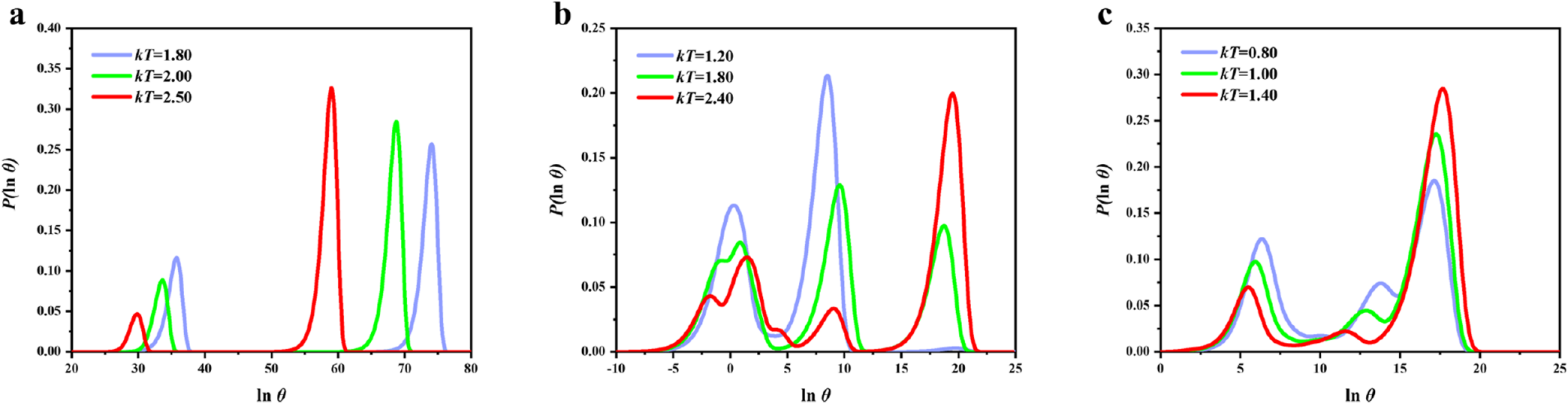
Examples of first passage time distributions for relaxation to the lowest minimum for (a) K_0_, (b) K_3_1__, and (c) K_7_1__. In each case the starting minimum was selected to highlight multiple relaxation times, and we show how the time scales change with the temperature. Here θ is the first passage time, i.e., the time taken for a trajectory to first reach the chosen product state from a given starting point. Plotting the probability distribution for the logarithm, P(ln⁡θ) , highlights separate relaxation times as different peaks.^22,127^

Figure 6 presents the disconnectivity graphs for the K_0_, K_3_1__, and K_7_1__ systems, respectively, each containing the 3000 lowest-energy local minima, as above. In these graphs, branches are colored according to the winding number of each minimum, and representative structures corresponding to the lowest minimum in each major funnel are shown at the bottom. For K_0_, although no topological knots are present, the landscape exhibits a clear multifunnel structure that can be roughly divided into a number of different regions. The two funnels containing the lowest minima on the left and right of the figure consist of structures that have uniformly small values for the winding number. This order parameter distinguishes several other funnels that have larger WN, corresponding to structures with multiple rotations around the center of geometry in a 2D projection, which mainly appear in the blue region of this spectrum. There is also a funnel with minima that have intermediate values of WN, where the branches are (mostly) green. Hence, we find that this parameter is quite successful in distinguishing families of minima that are also grouped together according to their mutual kinetic accessibility in the disconnectivity graph. The same is true for the other two topologies, despite the fact that the winding number does not correlate with the energy itself. This is an interesting observation, suggesting a connection between a measure of structural complexity (coiling), and the organization of the potential energy surface.

**Figure 6:**
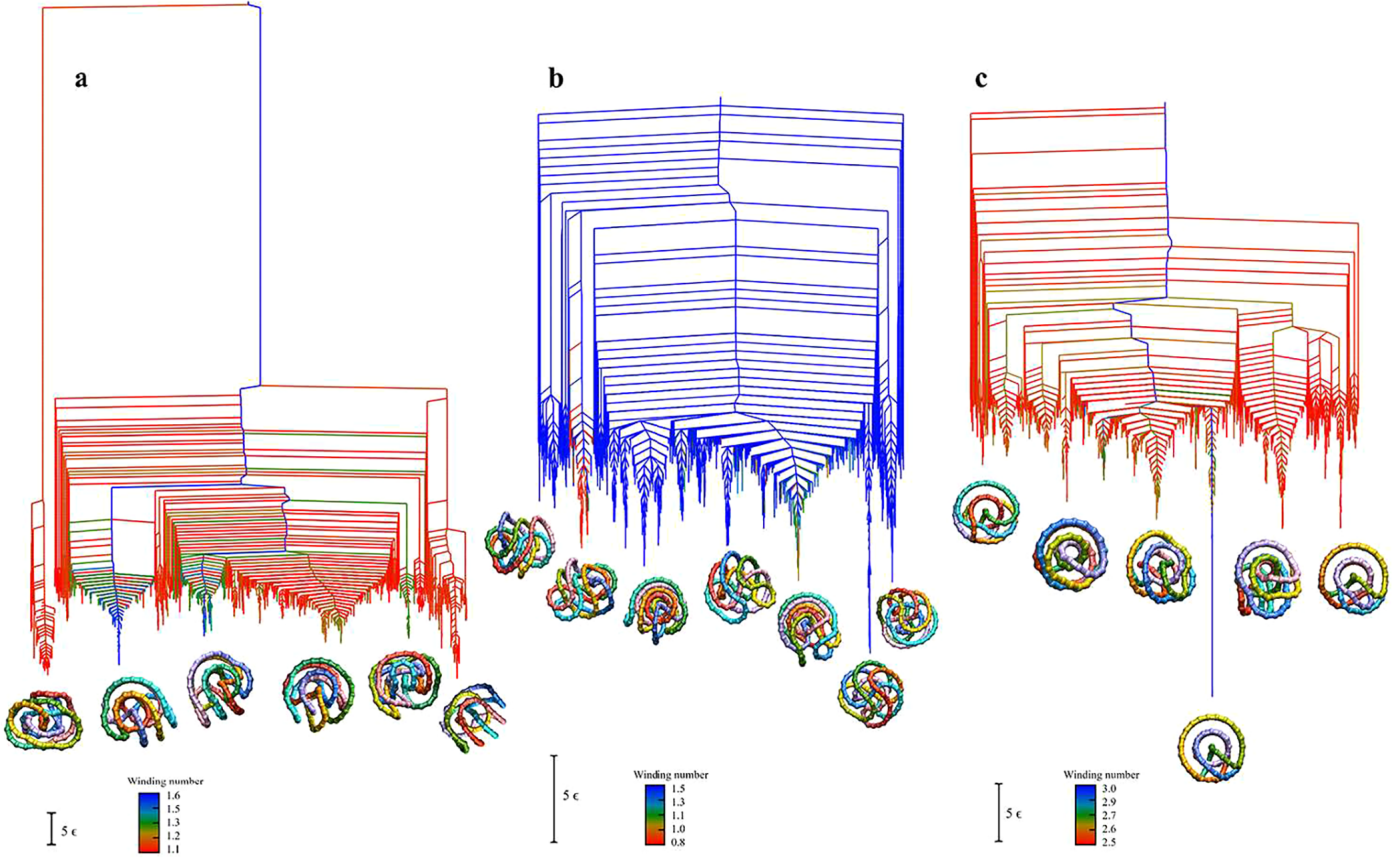
Disconnectivity graphs for the (a) K_0_, (b) K_3_1__, and (c) K_7_1__ landscapes, including only the lowest 3000 local minima.The branches are colored according to the winding number of each minimum, as indicated by the color scale (ranging from red to blue, corresponding to low to high winding degrees). The structures shown at the bottom represent the lowest-energy minimum from each funnel, providing a visual representation of the conformations associated with different basins.

In Figure 6b we see that the landscape for K_3_1__ is dominated by multiple funnels corresponding to relatively large winding number (approximately 1.5, in blue), including the lowest minimum. These funnels are energetically separated, but the minima have similar winding numbers, indicating that under the topological constraint of a trefoil knot, the system tends to adopt a series of compact, highly coiled low-energy conformations. As observed from the snapshots at the bottom of the disconnectivity graph, although the lowest-energy minima in these blue funnels share similar WN values, their structural morphologies differ significantly. The lowest 3_1_ minimum exhibits a flatter geometry with regular strand crossings and highly ordered spatial winding. In contrast, the remaining structures with similar WN can be broadly categorized into two types: one consisting of near-ring-like conformations with moderate bending, and the other displaying flattened conformations with less regular crossing patterns, generally less ordered than the lowest minimum. In addition to these dominant high-WN regions, the landscape also contains two distinct funnels. One is located on the right side of the graph and includes intermediate winding numbers (between around 1.05 to 1.1, colored green and yellow). The conformation of the lowest-energy minimum in this funnel appears more relaxed, exhibiting a bent, ring-like geometry. The other distinct funnel on the left side of the graph, contains minima with lower WN values (around 0.8, colored red). These structures correspond to more extended chain conformations with minimal spatial coiling and no prominent structural features. In summary, the K_3_1__ system exhibits a multifunnel landscape characterized mostly by minima with higher winding number, but there exist distinct alternative low-energy packings. Unlike K_0_, where low-energy conformations mostly correspond to smaller WN values, the trefoil knot topology promotes the formation of several stable, spatially coiled conformations with diverse folding geometries.

In Figure 6c we see that the global minimum for K_7_1__ which also has the highest observed mean winding number (approximately 3.0). This conformation exhibits point group symmetry *C*_2_ and a regularly coiled three-dimensional structure, featuring tight spatial winding and orderly strand crossings. In contrast, nearly all other local minima for K_7_1__ have smaller winding numbers (around 2.5), which is still larger than for the other topologies. As seen from the snapshots in the Figure, although these structures retain a certain degree of coiling, they have lower symmetry and less regular crossing patterns. In addition, a few local minima have intermediate WN values (around 2.6 to 2.7, colored green and yellow); they are scattered across the landscape and do not define local funnels. For this particular knot, the energy landscape becomes somewhat fragmented, with one particularly stable structure, and a number of alternative, higher energy, funnels. In summary, K_7_1__ exhibits a multifunnel energy landscape characterized by one high-WN, structurally ordered deep funnel, with several shallower funnel structures containing less ordered minima. It is worth noting that the lowest minimum for each of the three topologies exhibits a relatively high winding number and a geometrically regular coiled conformation, suggesting that this measure of structural complexity may be useful for understanding the organization of ring polymer molecules.

Figure 7 presents the corresponding disconnectivity graphs with the branches color-coded by the writhe value of each minimum, which quantifies the degree of spatial twisting and coiling between chain segments in three dimensions. For K_0_ (Figure 7a), the writhe values are intermediate between the ranges spanned by the other two topologies. Most conformations exhibit moderate spatial coiling, and only a few regions display significantly higher or lower writhe. These are the same regions that stand out for the winding number, above; the lowest minimum in these funnels has the highest writhe value (1.6, blue), and as the energy increases in this region the writhe decreases. Since the writhe is computed from multiple random directional projections by evaluating the over- and under-crossing relationships, it effectively captures the average degree of three-dimensional twisting in the chain structure, offering an alternative measure of spatial complexity compared to the winding number. Despite the absence of a topological knot, this ring system still features stable conformations with pronounced three-dimensional coiling. This observation suggests that geometric complexity can arise not only from topological constraints, but also from spontaneous conformational self-organization when sufficient degrees of freedom are available.

**Figure 7:**
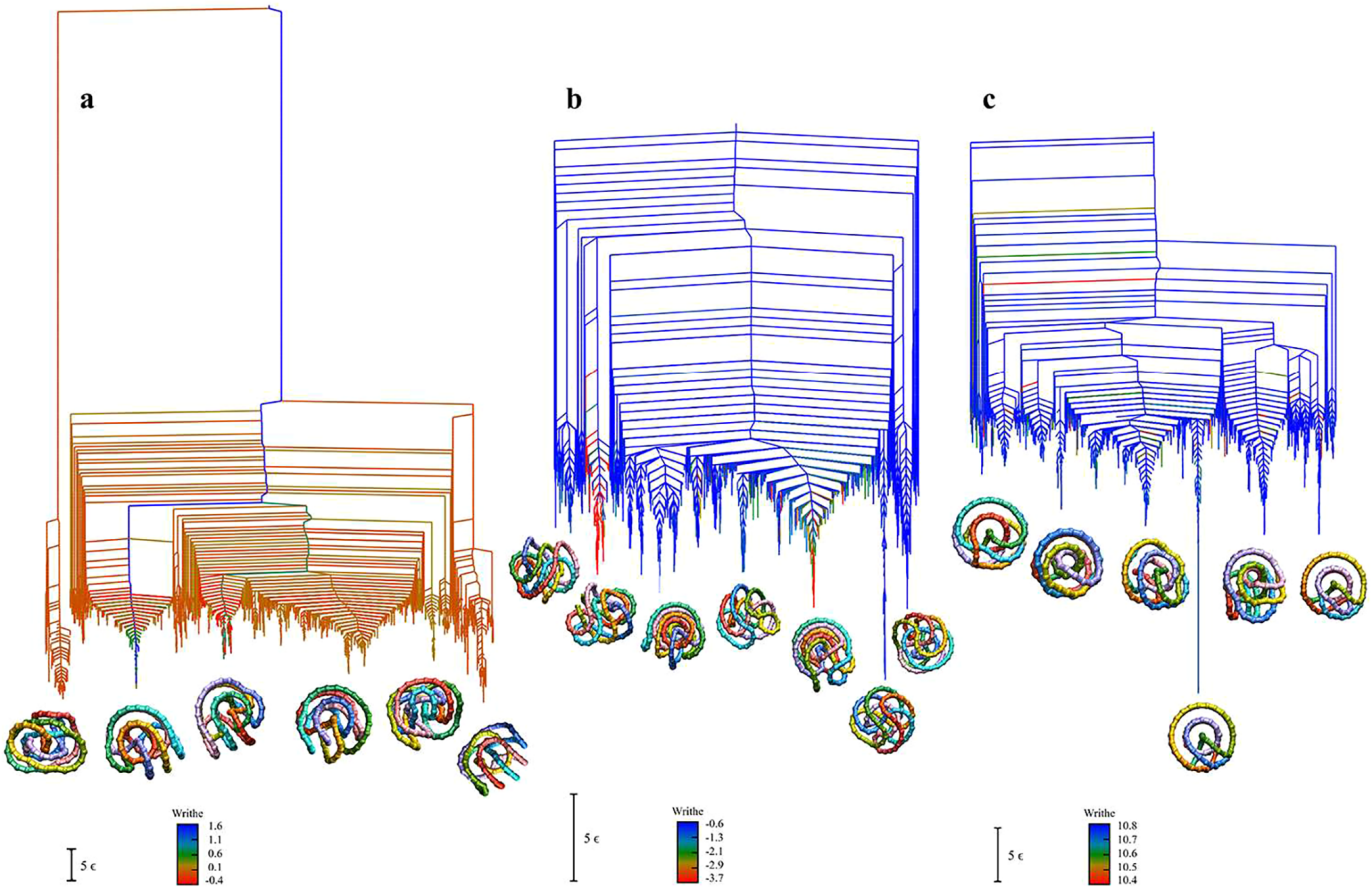
Disconnectivity graphs for the (a) K_0_, (b) K_3_1__, and (c) K_7_1__ landscapes, including only the lowest 3000 local minima. The branches are colored according to the writhe of each minimum, as indicated by the color scale (ranging from red to blue, corresponding to low to high writhe values). The sign of the writhe would be reversed for a right-handed 3_1_ topology; here we have chosen a left-handed molecule. The structures shown at the bottom represent the lowest-energy minimum from prominent funnels, providing a visual representation of the conformations associated with different basins.

Figure 7b presents the disconnectivity graph for K_3_1__. The writhe values in this system are negative for these minima because the trefoil knots present in the database are left-handed, as in Figure 1. Since writhe is a geometry- and direction-dependent quantity calculated from the signed crossings in two-dimensional projections, a left-handed 3_1_ knot under standard orientation yields negative writhe values. The handedness that we have sampled is arbitrary, and the same landscape would be obtained for either chirality. When we sample the databases of stationary points, we obtain connected minima with the same chirality, consistent with high chain crossing barriers between alternative handedness. The color scale is therefore inverted relative to the K_0_ and K_7_1__, with the largest magnitude values in red. We have presented the landscape for this choice of 3_1_ chirality to illustrate the effect. The majority of local minima have writhe values concentrated around −0.6, including the lowest minimum. Although the corresponding structure is compact and densely entangled based on visual inspection, the magnitude of the writhe is relatively small. The writhe depends not only on the number of crossings, but is also sensitive to the distribution of crossing signs. Highly symmetric conformations tend to exhibit mutual cancellation of positive and negative crossings across multiple projections, resulting in small-magnitude net writhe values. In contrast, conformations with more uniform coiling directions may involve fewer crossings but yield larger absolute writhe values due to consistent sign accumulation. This behavior contrasts sharply with K_7_1__, where all the low-lying minima have much larger values for this parameter. These observations suggest that writhe captures different conformational features under distinct topological constraints: in the unknotted system, it directly reflects geometric compactness, whereas in topologically constrained systems, writhe is more indicative of the directionality and correlation of spatial coiling, rather than simply the degree of folding.

Two funnels stand out with larger magnitude writhe values (around −3, colored red to yellow), and they are again the same funnels distinguished by the winding number, above. The conformations with the largest magnitude for the writhe correspond to more expanded structures with relatively few strand crossings and less spatial coiling.

Figure 7c presents the disconnectivity graph for K_7_1__, where all the writhe values for the lowest minima are large and fall within a narrow range, indicating similar degrees of spatial coiling. Here the writhe values are positive, corresponding to a right-handed choice for the 7_1_ system. Writhe values were obtained by averaging over 10,000 random directional projections, so this chiral bias is meaningful. As a result, the sign of the writhe reflects not only the degree of geometrical twisting, but also captures the overall rotational correlation of the chain in three-dimensional space. This high degree of writhe uniformity suggests that, for this topological constraint, the system favors conformations with consistent coiling direction, resulting in a coherent three-dimensional winding character throughout the low-energy conformational space. The structures illustrated at the bottom further support this observation: the lowest-energy minima from different funnels exhibit similar geometries, all adopting compact and regular spiral-like structures. The existence of a particularly favorable, higher symmetry, packing for the 7_1_ topology probably produces this more homogeneous landscape in terms of the writhe, even though the landscape itself has multifunnel character.

## IV Conclusions

The energy landscapes of 100-particle ring polymer molecules K^100^ have been explored by construction of kinetic transition networks, and analysis of how thermodynamic and kinetic properties are encoded. Three different topologies have been compared in detail, including K_7_1__ with crossing number 7, which supports the global minimum. Each landscape is multifunnelled, but the associated relaxation time scales vary over a remarkably wide range.

Structure prediction via global optimization restricted to a particular topology necessitates geometry perturbations that do not allow chain crossing events. We therefore introduced perturbations based on a radial potential in the basin-hopping global optimization process, which facilitates transitions between neighboring local minima while preserving the topological class. Moreover, the same potential provides a scheme for straightforward determination of crossing numbers in complex structures, where conventional approaches fail because there is no projection where the number of crossings is small. However, if we expand the collapsed structure using a suitable radial potential the crossing number becomes clear. The radial potentials preserve the topology within a wide range of parameter choices, and this approach also works for the linked and composite knots we have tested.

We further analyzed the energy landscapes in terms of the observable thermodynamic and kinetic properties. The heat capacity curves, *C*_*V*_, reveal distinct transition features. The K_7_1__ system exhibits the largest heat capacity peak, which also appears at a higher temperature than for K_0_ and K_3_1__. The corresponding landscape exhibits a particularly favorable, low-energy minimum, with *C*_2_ point group symmetry and a large heat capacity feature where the system starts to explore a wider range of structures that are significantly higher in energy. In contrast, the main *C*_*V*_ peak of K_0_ appears at lower temperature, and involves fewer minima. In this case there are two competing structures with similar energy, but qualitatively different packing schemes.

The multifunnelled character of each landscape is clearly reflected in the global kinetics. Here we calculated the mean first passage time (MFPT) to the lowest minimum with the same topology in each case, using the graph transformation approach, which can treat highly ill-conditioned problems with long time scales. The MFPT segregates the landscapes very clearly according to the overall barrier heights. The K_0_ system exhibits a remarkably deep kinetic trap, and corresponding broken ergodicity. The fastest path from the trap to the lowest K_0_ minimum involves approximately 630 transition states, with a barrier exceeding 700 *k*_B_ *T* at the temperature of the heat capacity peak, and involves collective reorganization rather than a single rate-limiting step. The MFPT analysis further reveals differences in relaxation behavior across the topologies: K_7_1__ structures relax most efficiently to the lowest minimum, followed by K_3_1__, while K_0_ has much longer time scales. We have also calculated first passage time distributions, which highlight the wide range of relaxation time scales associated with the array of kinetic traps. We would expect these time scales to be evident in time-resolved experiments that interrogate alternative morphologies. The present approach could be applied to models of specific experimental systems in the future.

We find that measures of knotting complexity, especially the winding number and writhe, provide order parameters that successfully identify families of structures in particular funnels. Hence there are correlations between the complexity and the organization of the landscape in terms of local kinetic accessibility. These order parameters therefore provide insight that is not available by visual inspection of structures for similarity. Understanding such correlations between quantitative measures of geometrical complexity and observable molecular properties is a promising direction for future research.

The different topologies would not interconvert for strongly bonded particles under experimental conditions, unless the bonds could be broken. We have identified cases where low-energy structures are predicted to be trapped behind insurmountable barriers; for K_0_ the corresponding paths involve large-scale reorganization requiring hundreds of transition states. If these morphologies could be realized experimentally they would provide alternative building blocks for polymeric materials that would have distinct physical characteristics. Understanding these building blocks and their properties at an atomistic level of detail could provide design principles for such materials. We have already found that the crystallization behavior in bulk simulations exhibits systematic differences for the three K^100^ topologies we have considered. These results will be presented in a future report.

## Supplementary Material















## Data Availability

The GMIN,^87^ OPTIM,^88^ and PATHSAMPLE^89^ software is freely available under the GNU public license at https://www-wales.ch.cam.ac.uk/software.html.

## Notes

Classification of topologies using the radial expansion potential; energy as a function of integrated path length for K_7_1__^100^ as a function of the σ and ε parameters in the Lennard–Jones potential (PDF)
K100.0.expand (MPG)
K100.3_1.expand (MPG)
K100.0.expand (AVI)
K100.7_1.expand (MPG)
K100.3_1.expand (AVI)
K100.7_1.expand (AVI)
