# Supplementary material for: Energy Landscapes of Model Knotted Polymers
Source: J Chem Theory Comput. 2025 Aug 14;21(16):8168–81. doi: 10.1021/acs.jctc.5c01005 (PMC12392451; doi:10.1021/acs.jctc.5c01005)
Supplement: Supplementary file 1 [file ct5c01005_si_001.pdf]

Energy Landscapes of Model Knotted Polymers: Supplementary information

Tongfan Hao,[1,2] Yinghao Ge,[1] Mark A. Miller,[3] Agustin L. N. Francesco,[2] and David J. Wales[2,*]

[1]*School of Materials Science and Engineering,*
Jiangsu University, 301 Xuefu Road, Zhenjiang 212013, China

[2]*Yusuf Hamied Department of Chemistry, University of Cambridge,*
Lensfield Road, Cambridge CB2 1EW, UK

[3]*Department of Chemistry, Durham University, South Road, Durham DH1 3LE, UK*

(Dated: August 1, 2025)

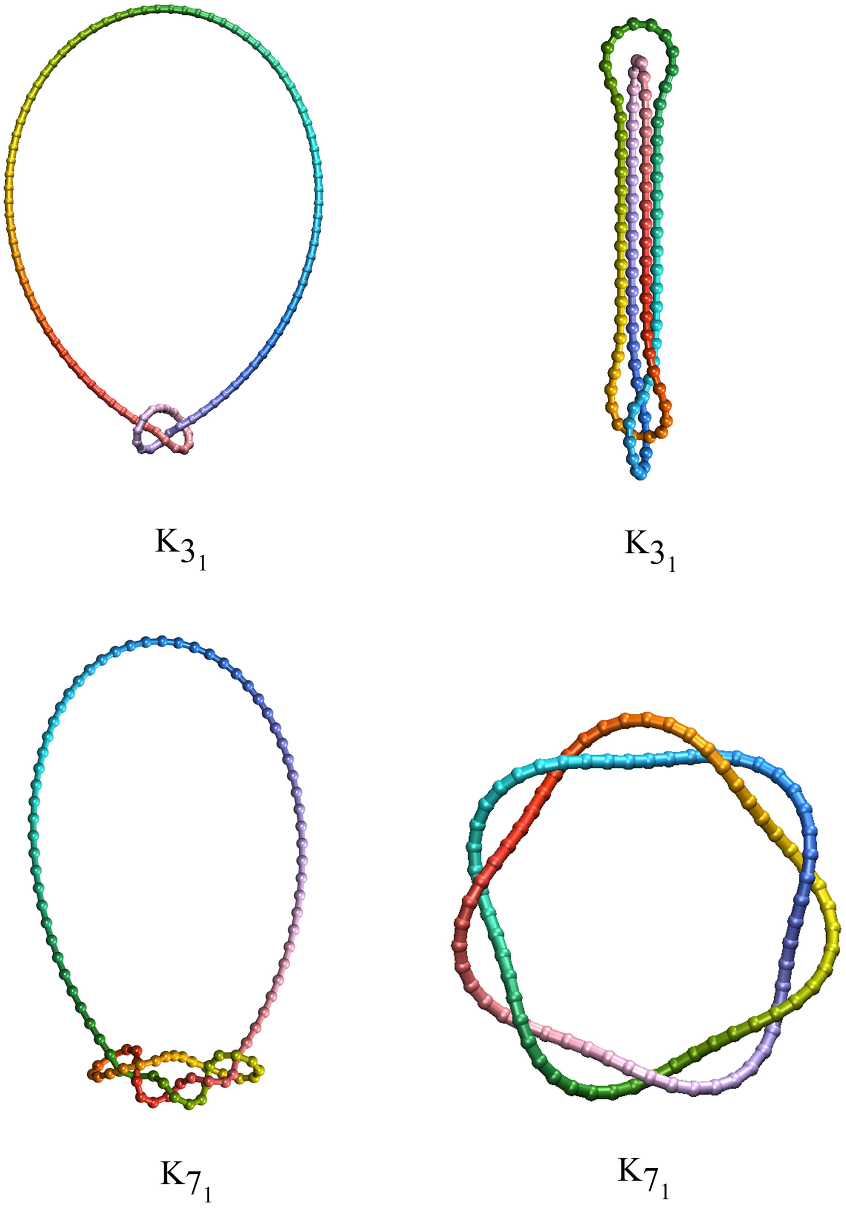

FIG. 1. Classification of topologies using the radial expansion potential. Here we illustrate the two different minima identified for $K_{3_1}^{100}$ and $K_{7_1}^{100}$ in surveys over a wide range of the parameter k in equation (3). In each case the two minima coexist for a range of k, and the localised crossings for the structures on the left appear when k is larger in magnitude (more negative).

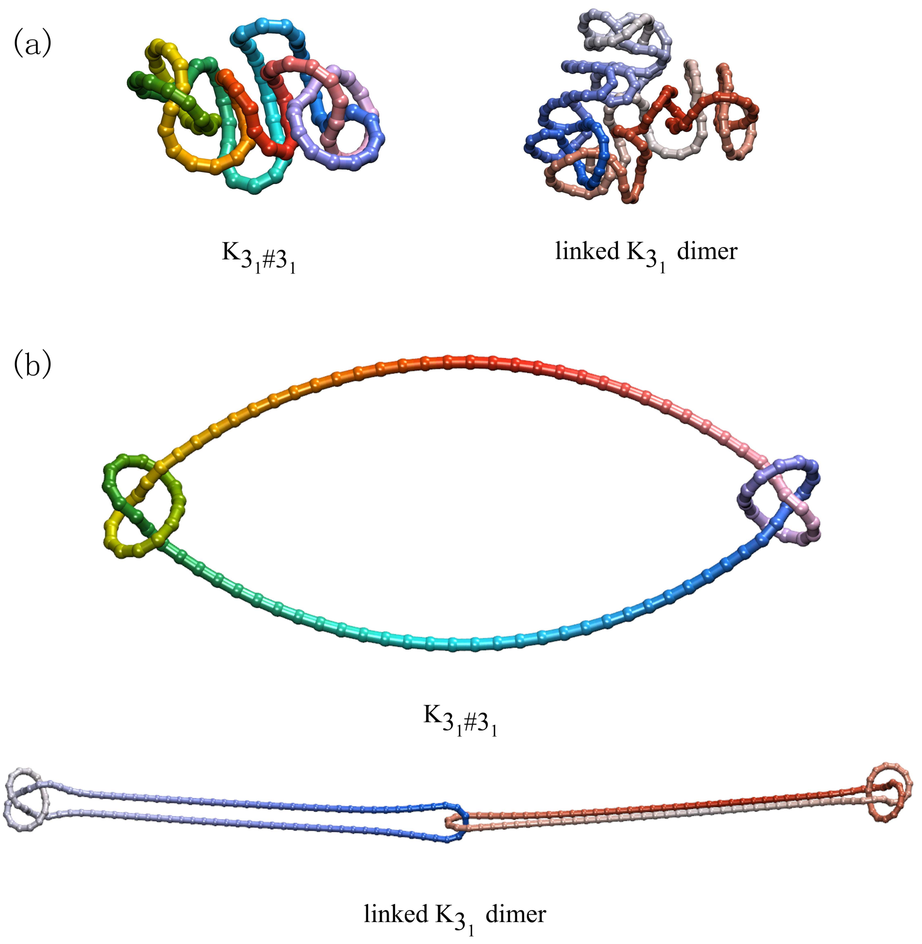

FIG. 2. Compact (a) and expanded (b) minima for the $K_{3_1}^{100}$ dimer link and for the $K_{3_1\#3_1}^{100}$ composite knot, where we see that the radial potential factorises the composite into its primes.

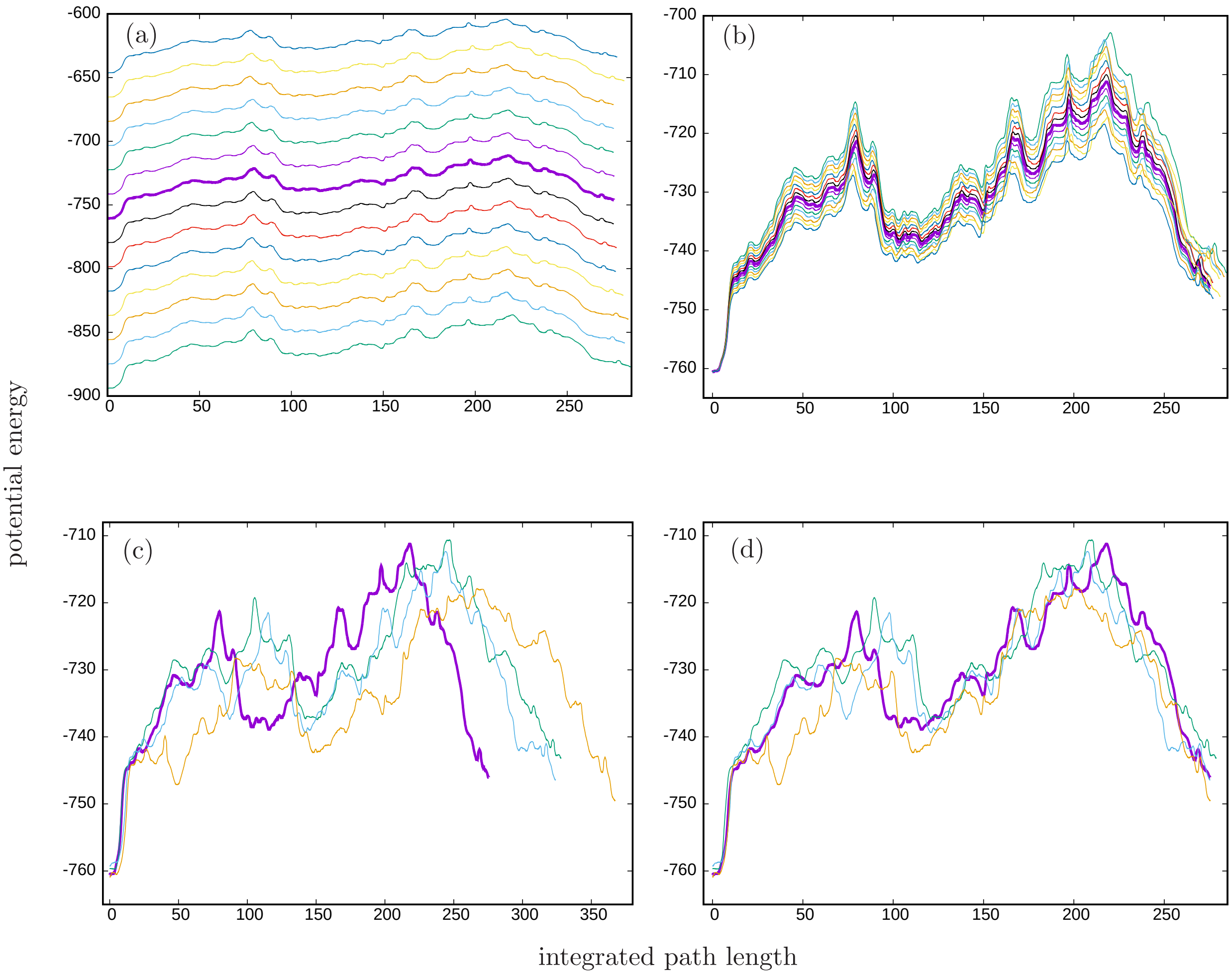

FIG. 3. Energy as a function of integrated path length for $K_{7_1}^{100}$ as a function of the σ and ε parameters in the Lennard-Jones potential equation (1). The path for the original parameters, $\varepsilon = 1$ and $\sigma = 1.888$, links the global minimum and the minimum at the bottom of the next-lowest energy funnel in 23 steps (transition states). It corresponds to the largest contribution to a steady-state rate constant, identified using the Dijkstra algorithm[1] with appropriate edge weights[2] at a temperature of $k_B T = 1.628$. This path is highlighted as the thickest line in all four panels. All the stationary points and pathways for the different parameters are fully reoptimised. (a) Energy profiles as ε is changed between 0.85 (highest energy) and 1.175 (lowest energy) in steps of 0.025. (b) The profiles in panel (a) are shifted vertically to align the global minimum. Each step of 0.025 in ε changes the global minimum energy by approximately 19 units. (c) Energy profiles for σ increased by 1% and decreased by 1% and 2%, shifted on the vertical axis to align the global minimum. (d) As for panel (c) with the integrated path length scaled to give approximately the same displacement between the end minima.

---

* dw34@cam.ac.uk

[1] Dijkstra, E. W. A note on two problems in connexion with graphs. *Numerische Math.* **1959**, *1*, 269–271.

[2] Wales, D. J. Discrete path sampling. *Mol. Phys.* **2002**, *100*, 3285–3305.
